# Human Papillomavirus Infection and Vaccination: Knowledge and Attitudes among Nursing Students in Italy

**DOI:** 10.3390/ijerph16101770

**Published:** 2019-05-19

**Authors:** Concetta Paola Pelullo, Maria Rosaria Esposito, Gabriella Di Giuseppe

**Affiliations:** 1 Department of Experimental Medicine, University of Campania “Luigi Vanvitelli”, 80138 Naples, Italy; concettapaola.pelullo@unicampania.it; 2 Istituto Nazionale Tumori, “Fondazione G. Pascale”, 80131 Naples, Italy; m.rosiespo@gmail.com

**Keywords:** attitudes, cross-sectional study, HPV infection, knowledge, nursing students, vaccination

## Abstract

This cross-sectional study assessed nursing students’ knowledge and attitudes about Human papillomavirus (HPV) infection and vaccination in Italy. The survey was conducted among a sample of 556 nursing students. Almost all reported that they had heard about HPV infection, while only 36.5% knew the risk factors of HPV infection and that this could be prevented by the HPV vaccine. Those who had heard about HPV infection during their degree program were more likely to know risk factors of HPV infection and that this could be prevented by the HPV vaccine. The majority of students (65.3%) reported that they would be willing to receive the HPV vaccine. Moreover, 91.7% of participants reported that they were willing, as future health care operators, to recommend the HPV vaccine to others. Those who knew risk factors of HPV infection and that this could be prevented by the HPV vaccine, and those who knew that cervical cancer could be prevented by the HPV vaccine expressed this positive attitude about willingness to recommend the HPV vaccine. These results highlight the need to supplement nursing students’ specific education, to improve their knowledge and awareness of HPV vaccination.

## 1. Introduction

Human Papilloma virus (HPV) infection is one of the most frequent sexually transmitted infections. In particular, some genotypes can cause cervical cancer and, in addition, they can be responsible for other cancers, such as anal (90%), vaginal (70%), penile (50%), vulvar (40%) and oropharyngeal (26%) [1]. According to the most recent data from National Institute of Health, in Italy, 1515 new cases of cervical cancer were diagnosed and 697 deaths were attributed to this type of cancer [2].

Since 2008, the HPV vaccine has been included in Italy’s national vaccination program, where it is offered to girls aged between 11 and 12 years [3]. Moreover, the recent National Vaccine Prevention Plan 2017–2019 extended the vaccination program to include young men [4]. However, using data collected by the end of 2017, it is estimated that 71.5% and 74.2% of girls aged 12–16 years and aged 13–20 years, respectively, had received at least one dose of HPV vaccine, while 19% of boys aged 12–14 years had received it. The levels of vaccine coverage are low compared to the ≥60% in males and ≥95% in girls coverage target [5].

To encourage higher HPV vaccine uptake, the general population requires accurate information about the utility of the vaccine in HPV infection prevention. Indeed, the achievement of vaccinations is related to the awareness of health professionals on this topic. Therefore, health care workers play an important role in health promotion via the provision of correct, complete and comprehensible information. Furthermore, in addition to physicians, other health care workers such as midwives and nurses must be adequately trained in order to offer, depending on their role, appropriate information consistent with maximizing adherence to vaccination and, they are an important population to study their level of knowledge, attitudes and behavior regarding HPV infection and related vaccination [6].

In the literature, several studies have focused on the attitudes and behaviors of at risk groups [7,8,9,10,11] and physicians [12,13,14,15,16] about HPV infection and vaccination, while a few studies have included nurses [17,18,19] or nursing students [20,21,22]. The purpose of this study was to assess the level of knowledge and attitudes about HPV infection and vaccination among a random sample of nursing students attending Italian universities as well as to identify the factors associated with the main outcomes of interest.

## 2. Materials and Methods

This cross-sectional study was conducted in a random sample of 556 nursing students (attending first, second and third year of training) who were recruited between February and April 2018, in three Italian universities, two in Campania and one in the Lazio region. In Italy, access to nursing courses is restricted, and all students must pass an exam prior to enrolment. The degree course consists of three years of academic education and is considered as an undergraduate degree program in nursing. The sample was selected using a two-stage cluster method. In the first stage, from a list of all the degree programs in nursing in each University, five degree programs in nursing were randomly selected. Subsequently, a random sample technique was used to recruit nursing students.

The directors of each selected school for nursing, before giving their approval, received a letter outlining the study. The survey tool was a self-administered anonymous and structured questionnaire. In each course, an interviewer, gave instructions about the questionnaire to the nursing students, guaranteed the confidentiality of the information provided and the anonymity of all data. Informed consent for participation was requested before completing the questionnaire.

The HPV questionnaire was based on a survey implement used in a previously published study, which assessed HPV infection and vaccination related knowledge, attitudes and behavioral intentions among adolescents and young women [7]. Two researchers reviewed the questionnaire prior to administration and subsequently adapted it for the nursing students.

The questionnaire comprised the following five sections: (1) socio-demographic characteristics (age, gender, marital status, number of cohabiting, parents’ educational level and parents’ working activity); (2) knowledge of HPV infection and vaccination; (3) attitudes towards HPV infection and vaccination (perception of risk towards HPV infection and towards HPV-related diseases, usefulness of the vaccine in men and women and safety of the vaccine); (4) willingness to receive the HPV vaccine in the future; and (5) need of information about HPV vaccine. Attitudes towards HPV infection and vaccination were measured on a 10-point Likert scale ranging from 1 (not worried) to 10 (extremely worried). Willingness to receive the HPV vaccine in the future was also measured on a 10-point Likert scale ranging from 1 to 10, with a higher score indicating a greater intention to be vaccinated. For each response, the respondents explained their reasons. (S1: Questionnaire)

Before study initiation, a sample size was calculated assuming a prevalence rate of 86% of nursing students who have heard about HPV, using a confidence interval of 95%, a margin of error of 5% and a design effect of 2. Finally, assuming a response rate of 95%, the total required sample size was 389 nursing students.

The pilot study was conducted with a sample of 50 respondents, included in the final sample, to evaluate the comprehensiveness and readability of the survey instrument.

Prior to the study initiation, the approval of the Ethics Committee of the University of Campania “Luigi Vanvitelli” was obtained (No 59 13 February 2018).

### Statistical Analysis

In this study, there were four outcomes of interest: (a) knowledge of risk factors (sexual intercourse at an early age, high frequency of sex partner exchange, multiple sexual partners and unprotected sex with partners) of HPV infection and that this could be prevented by HPV vaccine (no = 0; yes = 1), (Model 1); (b) positive attitude towards the safety of the HPV vaccine (no = 0; yes = 1), (Model 2); (c) willingness to receive the HPV vaccine (no = 0; yes = 1), (Model 3); (d) willingness, as future health care operators, to recommend the HPV vaccine (no = 0; yes = 1), (Model 4). In all the models, the independent variables included were: gender (male = 0; female = 1), age (three categories: ≤20 years; 21–25 years; >25 years), educational level of at least one parent (others = 0; college degree or higher = 1), at least one parent is a health care operator (no = 0; yes = 1), have heard about HPV infection during their degree program (no = 0; yes = 1). The following variables were also included: need of additional information about HPV vaccine (no = 0; yes = 1) in Model 1; knowledge of risk factors of HPV infection and that this could be prevented by HPV vaccine (no = 0; yes = 1), knowledge that cervical cancer could be prevented by HPV vaccine (no = 0; yes = 1) and knowledge that men and women should be vaccinated (no = 0; yes = 1) in Models 2, 3 and 4; perception of risk of contracting HPV infection (0 = 1–5; 1 = 6–10), perception of risk of developing diseases caused by HPV (0 = 1–5; 1 = 6–10), perception of the usefulness of the HPV vaccine in protecting men and women (0 = 1–9; 1 = 10) and positive attitude towards the safety of the HPV vaccine (0 = 1–9; 1 = 10), in Models 3 and 4.

Stepwise logistic regression analysis was performed to evaluate the associations between the independent variables and the dichotomous outcomes of interest. Variables were selected for the multivariate model using a *p*-value of 0.2 for entry and 0.4 for exclusion, based on the likelihood ratio test statistic. Odds ratios (ORs) and 95% confidence intervals (CIs) are presented for logistic regression models; two-sided *p*-values ≤ 0.05 were considered to be statistically significant. Stata statistical software, version 15 (College Station, TX, USA) was used for the data analysis [23].

## 3. Results

556 nursing students returned the self-administered questionnaires, giving a response rate of 100%. Table 1 shows the respondents’ socio-demographic characteristics. The average age of the participants was 22.3 years (18–46 years), 65.5% were females and almost all were unmarried. Only 15.7% of the students were employed and 18.4% had at least one parent who was a health care operator.

### 3.1. Knowledge of HPV Infection

Almost all the students (96%) reported that they had heard about HPV infection, 30.8% by physicians and 64.9% during their degree program, while 85.6% knew that men and women could contract HPV infection. In terms of knowledge about diseases caused by HPV infection, the values in descending order were cervical (84.7%), penile (31.1%), oropharyngeal (23.9%) and anal (19.2%) cancers, and genital warts (29.9%) (Table 2).

Among the respondents, 36.5% knew risk factors of HPV infection and that this could be prevented by the HPV vaccine. The results of the multivariate logistic regression model showed that those who had heard about HPV infection during their degree program (OR = 2.15; 95% CI 1.43–3.23) were more likely to know risk factors of HPV infection and that this could be prevented by HPV vaccine. Moreover, this knowledge was significantly lower in nursing students under 20 years of age (OR = 0.56; 95% CI 0.36–0.86) compared to the age group >25. More than two-third (76.8%) knew that cervical cancer could be prevented by HPV vaccine and 53.6% were aware that both men and women should be vaccinated (Table 3).

### 3.2. Attitudes and Behavior about HPV Vaccination

The mean values for the perceived personal risk of contracting HPV infection and of developing diseases caused by HPV, measured on a 10-point Likert scale, were 4.3 and 4.4, respectively. The mean value for students’ attitudes to the usefulness of the vaccination in men and women, measured on a 10-point Likert scale ranging from 1 to 10 with higher scores indicating high utility, was 6.9 for HPV vaccine for men and 8.4 for women, with 28.1% reporting a very positive views, responding 10, for vaccination in both men and women (Table 4).

In terms of HPV vaccine safety, 20.5% considered it very safe, with a mean total score of 7.4. The multivariate logistic regression showed that those that knew the risk factors of HPV infection and that this could be prevented by the HPV vaccine (OR = 1.6; 95% CI 1.03–2.48), those that have heard about HPV infection during their degree program (OR = 1.72; 95% CI 1.05–2.83), those that knew cervical cancer could be prevented by HPV vaccine (OR = 2.11; 95% CI 1.16–3. 83) and those that knew that men and women should be vaccinated (OR = 1.57; 95% CI 1.02–2.43) were more likely to think that the HPV vaccine is very safe. Moreover, female participants (OR = 0.62; 95% CI 0.4–0.97) were less likely to think that HPV vaccine is very safe (Table 3).

Only 23.9% of respondents reported having undergone HPV vaccination. The majority of these respondents (88.7%) were females aged ≤20 years. In relation to intentions to be vaccinated against the HPV, 65.3% of nursing students responded ‘‘yes’’ on being asked about willingness to receive it. The results of the multivariate logistic analysis showed that those who were more willing to undergo future HPV vaccine were female (OR = 2.26; 95% CI 1.49–4.65), and those who considered HPV vaccine useful (OR = 2.64; 95% CI 1.49–4.65). Furthermore, students with age ≤20 years (OR = 0.58; 95% CI 0.34–0.98) were less likely to undergo future HPV vaccine compared to the age group >25 (Table 4) Moreover, the main reasons participants were not willing to believe that the vaccine is not useful (38.1%), out of fear (19%), not feeling at risk (15.9%) and not having it recommended by their physician (14.3%).

Almost all the respondents (91.7%) were willing, as future health care operators, to recommend the HPV vaccine to others. The results of multivariate logistic analysis showed that those who knew risk factors of HPV infection and that this could be prevented by HPV vaccine (OR = 3.11; 95% CI 1.27–7.66), those who knew that cervical cancer could be prevented by HPV vaccine (OR = 1.99; 95% CI 1.02–3.89) and those who heard about HPV infection during degree program (OR = 1.86; 95% CI 0.97–3.54) were more willing to recommend the HPV vaccine as future health care operators (Table 3).

### 3.3. Sources of Information

In terms of the need for additional information about the HPV vaccine, 88.5% of nursing students declared a need for additional information, particularly from physicians (53.6%) and during their degree program in nursing (55.4%).

## 4. Discussion

To the best of our knowledge, this is the first study conducted in Italy on nursing students’ knowledge and their attitudes towards HPV infection and vaccination. This population was selected because nurses play an important role, as health care operators, in health promotion. There is a need to implement knowledge and encourage positive attitudes towards prevention during nursing degree courses. A comparison of the results of this study with those of similar studies is difficult due to the scarcity of studies with the same aims or population.

In this survey, almost all the students have heard about HPV infection. The majority of the students also knew that the HPV can cause cervical cancer. The provision of information on HPV during their degree program in nursing may explain this finding. The level of student knowledge in this study was higher than that found in another study, where 89.3% of students in the health sector have heard about HPV infection [24]. Moreover, 84.7% of students in the present study knew that HPV infection could cause cervical cancer. This knowledge was higher than that observed in two studies conducted in Turkey and one in India [20,21,24]. In contrast, a higher level of knowledge was observed in studies performed in USA (90.5%) and United Kingdom (90.4%) [22,25]. With regard to other cancers associated with HPV infection, in our study, only 31.1%, 23.9% and 19.2% of students knew that it could cause, respectively, penile, oropharyngeal and anal cancers, while higher rates have been reported in a previously mentioned study conducted in Turkey [22]. Thus, despite the high level of knowledge about HPV reported in this study, there was a lack of knowledge about diseases caused by HPV infection. In particular, only 29.9% of students knew that HPV could cause genital warts. Meanwhile, a higher level of knowledge about genital warts has been observed in other studies conducted in New Zealand and United Kingdom where, respectively, 89% and 78.7% of nursing students indicated correct knowledge about this finding [17,25]. These results are probably due to the fact that these countries have higher HPV vaccine uptake compared to Italy [26,27,28]. Indeed, in other studies, although with different population, HPV vaccine uptake has been associated, with higher HPV knowledge [29,30]. Furthermore, 76.8% knew that cervical cancer could be prevented by HPV vaccine. This finding was similar to that of another study, where 77.6% of nursing students indicated that cervical cancer could be prevented with vaccination [20]. In contrast, a higher level of knowledge was observed in a previously mentioned study, wherein 81.1% of students knew that HPV vaccine offer protection against most cervical cancer [17]. In order to increase uptake of the HPV vaccine, there is a need for correctly informing the population about the utility of prevention. Nevertheless, the quality of the information must be precise and clear to avoid misunderstandings [6].

In addition to HPV knowledge, we evaluated nursing students’ attitudes towards the HPV vaccine. In our study, 65.3% of students were willing to receive the HPV vaccine in the future and 91.7%, as future health care operators, to recommend the HPV vaccine to others. This finding can be compared with other studies. For example, Shetty et al. reported that 65.2% of students intended to receive HPV vaccine, while a lower level was found when asked about willingness to recommend the vaccine to others (68.3%) [24]. In contrast, a lower level of willingness towards HPV vaccination was found in a previously mentioned study, where 59.1% of student nurses indicated that they would like to have HPV vaccination for themselves and 77.2% to approve vaccination for others [20]. Moreover, also in another study conducted in Turkey 66.3% of nursing students were willing to be vaccinated [21]. Furthermore, Bal-Yilmaz et al. [22] reported that only 14.4% of nursing students intended to receive HPV vaccination.

In our sample, only 23.9% of nursing students reported having received the HPV vaccination. This finding is higher to two studies, the first conducted in India and the other conducted in Turkey, in which, respectively, nursing students who had undergone HPV vaccination were only 2.1% and 1.3% [21,22].

In our study, 62.9% of nursing students reported they had heard about HPV infection during degree program in nursing. Moreover, 55.4% of students stated that the degree program in nursing was one of their preferred sources of additional information about the HPV vaccine. This percentage was higher than that reported in another study, where only 42.1% of students reported that college and university were their main sources of information about HPV vaccination [24]. Moreover, in this survey, 53.6% of participants reported physicians as another important source for additional information about the HPV vaccine. This finding is higher to that highlighted in others studies [10,24].

This survey has some potential limitations. First, this was a cross-sectional study and it is difficult to determine the temporal relationship between the explanatory variables and the different outcomes of interest. Second, it is possible that the nursing students interviewed had favorable attitudes towards HPV vaccination and potentially were more likely to respond to the questionnaire. Third, this survey was based on a self-administrated questionnaire with self-reported information, thus participants could report correct behavior and not that which they deemed incorrect. Besides these limitations, the major strength of this survey was the high response rate, which means the results are representative of the population.

## 5. Conclusions

The success of the HPV vaccination program is closely related to awareness by health care professionals of the importance of this primary prevention intervention. Therefore, there is a need for education and information programs aimed at medical schools, particularly nursing degree courses, to increase health care operators’ knowledge about HPV infection and their awareness towards HPV vaccination. Moreover, future studies should address educational programs about HPV infection in nursing students and evaluate their effectiveness.

## Figures and Tables

**Table 1:** Socio-demographic characteristics about the study population.

	N	%
Age *	22.3 ± 3.4 (18–6) °	
≤20	155	28.1
21–25	332	60.3
>25	64	11.6
Gender		
Male	192	34.5
Female	364	65.5
Marital Status *		
Unmarried	505	97.1
Other	15	2.9
Employment Status *		
Employed	84	15.7
Unemployed	450	84.3
At Least One Graduate Parent *		
Yes	119	21.6
No	433	78.4
At Least One Health Care Worker Parent		
Yes	102	18.4
No	454	81.6

° Mean ± Standard deviation (Range), * Numbers may not add up to total number of study population due to missing values.

**Table 2:** Respondents’ knowledge of human Papilloma virus (HPV) infection and vaccination stratified for gender.

Knowledge	Total		Male		Female		
	N	%	N	%	N	%	*p*
Have Heard about HPV Infection							
Yes	537	96.6	181	33.7	356	62.3	0.029
No	19	3.4	11	57.9	8	42.1	
Risk Factors for HPV Infection							
Unprotected sex with partners (true)	523	94.1	177	33.8	346	66.2	0.312
High frequency of sex partner exchange (true)	491	88.3	163	33.2	328	66.8	0.187
Multiple sexual partners (true)	475	85.4	160	33.7	315	66.3	0.312
Sexual intercourse at an early age (true)	232	41.7	74	31.9	158	68.1	0.453
HPV Related Diseases							
Cervical cancer	471	84.7	149	31.6	322	68.4	0.001
Penile cancer	173	31.1	61	35.3	112	64.7	0.808
Genital warts	166	29.9	55	33.1	111	66.9	0.651
Oropharyngeal cancer	133	23.9	42	31.6	91	68.4	0.411
Anal cancer	107	19.2	38	35.5	69	64.5	0.812
Men and Women could Contract HPV Infection							
Yes	451	85.6	157	34.8	294	65.2	0.221
No	76	14.4	21	27.6	55	72.4	
Preventive Measures for HPV Infection							
Condom use in sexual intercourse (true)	520	95.2	175	33.7	345	66.3	0.105
Vaccination (true)	482	88.4	147	30.5	335	69.5	<0.001
Late start of complete sexual activity (true)	138	26.1	37	26.8	101	73.2	0.031
Late start of incomplete sexual activity (true)	92	17.5	29	31.5	63	68.5	0.740
HPV Vaccine can Prevent Cervical Cancer							
Yes	427	76.8	124	29	303	71	<0.001
No	129	23.2	68	52.7	61	47.3	

**Table 3:** Multivariate logistic analysis to characterize factors associated with different outcomes of interest.

Variable	OR ^●^	SE ^▪^	95% CI	*p* Value
**Model 1**. Knowledge of risk factors of HPV infection and that this could be prevented by HPV vaccine (no = 0; yes = 1)				
Log likelihood = −345.85, χ^2^ = 31.35 (3 df), *p* < 0.001 (sample size = 551)				
Having heard about HPV infection during their degree program	2.15	0.45	1.43–3.23	<0.001
Age				
≤20	0.56	0.12	0.36–0.86	0.009
>25	1.0 *			
Female	1.41	0.27	0.97–2.08	0.073
**Model 2**. Positive attitude towards the safety of HPV vaccine (no = 0; yes = 1)				
Log likelihood = −266.90, χ^2^ = 26.62 (9 df), *p* < 0.00016 (sample size = 548)				
Female	0.62	0.14	0.4–0.97	0.035
Knowledge that cervical cancer could be prevented by HPV vaccine	2.11	0.64	1.16–3.83	0.014
Knowledge of risk factors of HPV infection and that this could be prevented by HPV vaccine	1.6	0.36	1.03–2.48	0.037
Having heard about HPV infection during their degree program	1.72	0.43	1.05–2.83	0.031
Knowledge that men and women should be vaccinated	1.57	0.35	1.02–2.43	0.042
Age				
21–25	0.81	0.18	0.52–1.27	0.360
>25	1.0 *			
**Model 3**. Willingness to receive the HPV vaccine (no = 0; yes = 1)				
Log likelihood = −246.47, χ^2^ = 42.43 (8 df), *p* < 0.001 (sample size = 416)				
**Female**	2.26	0.51	1.46–3.51	<0.001
Awareness of usefulness of HPV vaccine	2.64	0.76	1.49–4.65	0.001
Knowledge that men and women should be vaccinated	1.38	0.31	0. 9–2.13	0.142
Age				
≤20	0.58	0.15	0.34–0.98	0.041
>25	1.0 *			
Positive attitude towards the safety of HPV vaccine	0.73	0.21	0.41–1.29	0.277
Knowledge that cervical cancer could be prevented by HPV vaccine	1.26	0.32	0.77–2.1	0.361
Having heard about HPV infection during their degree program	0.8	0.2	0.49–1.31	0.380
Perception of risk of contracting HPV infection	1.3	0.33	0.79–2.15	0.295
**Model 4**. Willingness, as a future health care operator, to recommend the HPV vaccine (no = 0; yes = 1)				
Log likelihood = −138.99, χ^2^ = 28.33 (7 df), *p* = 0.002 (sample size = 548)				
Knowledge of risk factors of HPV infection and that this could be prevented by HPV vaccine	3.11	1.43	1.27–7.66	0.013
Knowledge that cervical cancer could be prevented by HPV vaccine	1.99	0.68	1.02–3.89	0.043
Having heard about HPV infection during their degree program	1.86	0.61	0.97–3.54	0.06
Positive attitude towards the safety of HPV vaccine	2.96	1.82	0.88–9.9	0.078
Having undergone HPV vaccination	1.55	0.65	0.68–4.52	0.296
Perception of risk of developing diseases caused by HPV	1.77	1.02	0.57–5.49	0.320
Perception of risk of contracting HPV infection	0.59	0.33	0.19–1.78	0.348

* Reference category in multivariate analysis, ^●^ Odds Ratio, ^▪^ Standard.

**Table 4:** Attitudes regarding HPV infection and its vaccine.

Attitudes	Average
Perceived personal risk of contracting HPV infection	4.3 ± 2.5 (1–10) *
Perceived personal risk of developing diseases caused by HPV	4.4 ± 2.6 (1–10) *
Usefulness of the vaccination in men	6.9 ± 2.8 (1–10) *
Usefulness of the vaccination in women	8.4 ± 2.2 (1–10) *
Safety of HPV vaccine	7.4 ± 2.1 (3–10) *

* Mean ± Standard deviation (Range).

## Supplementary Materials

The following are available online at https://www.mdpi.com/1660-4601/16/10/1770/s1, S1: Questionnaire.
