# Supplementary material for: Human Papillomavirus Infection and Vaccination: Knowledge and Attitudes among Nursing Students in Italy
Source: Int J Environ Res Public Health. 2019 May 19;16(10):1770. doi: 10.3390/ijerph16101770 (PMC6572142; doi:10.3390/ijerph16101770)
Supplement: Supplementary file 1 [file ijerph-16-01770-s001.pdf]

S1. QUESTIONNAIRE

Section A.

Information about your socio-demographic characteristics.

A1. How old were you on your last birthday? ________

A2. Sex: □Male □Female

A3. What is your marital status? □ married □ single □ other ____________

A4. How many people live with you?_________

A5. What is the highest degree of education of your father?

A6. What is the highest degree of education of your mother?

A7. What is the occupation of your father?____

A8. What is the occupation of your mother?____

A9. What is your occupation?____

Section B.

Knowledge towards HPV infection and vaccination.

B1. Human papillomavirus (HPV) is a virus that causes a sexually transmitted infection, have you ever heard of it? (more than one answer is possible)

□ no □ yes, from whom? □ mass media □ degree program in nursing □ physicians □ internet

□ friends/family □ parents □ other_________________

B2. Who can contract HPV infection? □ only men □ only women □ men and women

B3. Which of these diseases are caused by HPV infection?

□ lung cancer □ cervical cancer □ penile cancer □ bowel cancer □ oropharyngeal cancer

□ anal cancer □ genital warts □ urinary tract infection

B4. What are risk factors for HPV infection?

	**Yes**	**No**	**I don't know**
Unprotected sex with partners	□	□	□
High frequency of sex partner exchange	□	□	□
Multiple sexual partners	□	□	□
Sexual intercourse at an early age	□	□	□

B5. Can HPV infection be prevented?

	**Yes**	**No**	**I don't know**
Condom use in sexual intercourse	□	□	□
Vaccination	□	□	□
Late start of complete sexual activity	□	□	□
Late start of incomplete sexual activity	□	□	□

B6. In Italy, vaccines against HPV infection are available, have you ever heard of them? (more than one answer is possible) □ no □ yes, from whom? □ mass media □ degree program in nursing □ physicians □ internet □ friends/family □ parents □ other________________

B7. Who should get the HPV vaccination? □ men □ women □ men and women □ I don't know

B8. Indicate, between following diseases, those that can be avoided with HPV vaccination?

□ lung cancer □ cervical cancer □ penile cancer □ bowel cancer □oropharyngeal cancer

□anal cancer □ genital warts □ urinary tract infection □ I don't know

Section C.

Attitudes towards HPV infection and vaccination.

C1. On a scale from 1 to 10, how much are you worried of contracting HPV infection? (1 not worried; 10 very worried)________

C2. On a scale from 1 to 10, how much are you worried of developing diseases caused by HPV? (1 not worried; 10 very worried)_______

C3. On a scale from 1 to 10, how useful do you believe HPV vaccination in men? (1 not useful; 10 very useful)__________

C4. On a scale from 1 to 10, how useful do you believe HPV vaccination in women? (1 not useful; 10 very useful) ________

C5. How safe do you believe HPV vaccination to be? (1 not safe; 10 very safe)_________

Section D

Behaviors towards HPV infection and vaccination.

D1. Have you been to a physician in the last year? □ no (**go to the question D3**.)□ yes

D2. Did physician tell you about HPV vaccination? □ no □ yes

D3. Did you receive HPV vaccination? □ yes (**go to the question D5**.) □ no

D4. Do you intend to receive HPV vaccination?

□ if yes, why?____

□ if no, why?____

D5. As future health care operator, do you recommend the HPV vaccine to others? □ no □ yes

Section E.

Sources of information about HPV vaccination.

E1. Do you feel you need more information about HPV vaccination? (more than one answer is possible)

□ no □ yes, from whom? □ mass media □ degree program in nursing □ physicians □ internet

□ friends/family □ parents □ other________________
